# Recent advances in nanomedicines for regulation of macrophages in wound healing

**DOI:** 10.1186/s12951-022-01616-1

**Published:** 2022-09-09

**Authors:** Alireza Joorabloo, Tianqing Liu

**Affiliations:** NICM Health Research Institute, Western Sydney University, Westmead, NSW 2145 Australia

**Keywords:** Nanomaterials, Macrophages regulation, Nanomaterial-macrophage interaction, Drug delivery, Wound healing

## Abstract

Macrophages are essential immune cells and play a major role in the immune response as pro-inflammatory or anti-inflammatory agents depending on their plasticity and functions. Infiltration and activation of macrophages are usually involved in wound healing. Herein, we first described macrophage polarization and their critical functions in wound healing process. It is addressed how macrophages collaborate with other immune cells in the wound microenvironment. Targeting macrophages by manipulating or re-educating macrophages in inflammation using nanomedicines is a novel and feasible strategy for wound management. We discussed the design and physicochemical properties of nanomaterials and their functions for macrophages activation and anti-inflammatory signaling during wound therapy. The mechanism of action of the strategies and appropriate examples are also summarized to highlight the pros and cons of those approaches. Finally, the potential of nanomedicines to modulate macrophage polarization for skin regeneration is discussed.

## Introduction

The ultimate goal of wound management is to accelerate the wound repair and restoration process with the reduction of pain, scarring, and bacterial infections while preserving tissue function. However, it is a complicated and dynamic physiological process. To overcome the setbacks of insufficient expression of cellular markers, nutrients, and angiogenesis factors that cause wound healing delay, various types of cellular mediators, growth factors, and proteinases are required [1–4]. Different from acute wounds which take only a short time to heal through the normal process of wound healing [5, 6], chronic wounds takes several months to heal or may never heal due to disrupted healing process [7–9]. Chronic wounds are often characterized by persistent inflammation, presence of necrotic tissue, and poor dermal matrix integrity, which can negatively impact wound repair. They are normally associated with pathological conditions of certain diseases (e.g. diabetes), infections, or inadequate vasculature. Some common examples include pressure ulcers, diabetic ulcers, venous ulcers, and arterial ulcers. The associated complications, such as neuropathy, hyperglycemia, even gangrene or sepsis, can lead to poor quality of life of patients and increased risk of amputation [10, 11]. In addition, as the population ages, there will be a significant increase in numbers. As chronic wounds impact a large proportion of the world population, the care of chronic wound has become a major global concern for the health organizations, resulting in enormous expenses [12, 13]. In order to meet the rising demands worldwide, it is critical to find for optimum treatment options to shorten recovery time, improve patient quality of life, and lower the health-care cost [14–16].

Wound healing process generally divided into three continuous and overlapping phases, including inflammation, proliferation, and tissue remodeling [17, 18]. Macrophages are essential immune cells actively involved in the wound regeneration and the key regulators to ensure proper healing and tissue regeneration. The dynamic and plastic nature of macrophages allow them to change with local micro-environmental stimuli or signals [19–21]. They can be polarized into two distinct phenotypes, that is, pro-inflammatory macrophages (also called classically activated M1 phenotype) and anti-inflammatory macrophages (also called alternatively activated M2 phenotype). M1 macrophages (around 85%) at the initial stage infiltrates to the wound site to clean dead cells, debris, and bacteria. After about 7 days post injury, transition of macrophages from M1 phenotype to M2 phenotype (around 80–85%) is activated to promote anti-inflammatory effects, innate immunity, and tissue formation by proliferation of fibroblasts, keratinocytes, and endothelial cells in order to restore skin layers properly [22, 23]. In addition, macrophages secrete matrix metalloproteinases (MMPs) to transform temporary form of extracellular matrix (ECM) to original one at the final remodeling stage [24]. Therefore, in chronic wounds where pro-inflammatory macrophages resist converting to anti-inflammatory ones and tissue repair is impaired, it is essential to induce macrophages with favorable phenotypes for the treatment of wounds [18, 23, 25, 26].

Macrophage-based therapy has been introduced as a promising approach in chronic wound treatment [27, 28]. Currently, methods, including administration of activated macrophages, use of cytokines, dressings loaded with macrophage-modulatory therapeutic agents, and design of biomaterials or nanomedicines to manipulate macrophage infiltration or macrophage phenotypical differentiation, are utilized to engineer macrophages [29, 30]. However, more efforts are needed to overcome the limitations that lead to chronic wound treatment failure, including (1) the formation of biofilms at the wound site due to limited antibacterial activity of wound dressings, (2) lack of physical and mechanical properties of wound dressing materials such as low water vapor transmission rate, poor wound fluid absorption capacity, low porosity, and non-breathability, (3) low biocompatibility and physicochemical instability that may cause the degradation or deactivation of drugs or biological therapeutic agents such as growth factors, and (4) low level of angiogenesis, poor pharmacokinetics, and chronic inflammation that were not addressed by traditional wound cares [31–35]. Nanomedicines have been hailed as a huge success when it comes to treat chronic wounds by providing excellent physicochemical properties for the dressing [36–38], increasing the performance of antibacterial activity by prevention of biofilm formation [39–41], maintaining the stability of the loaded growth factors or drugs [42, 43], and improving cell functions and microenvironment for wound closure [44]. More recently, immunomodulatory bionanomatierals have been emerged as a powerful tool to enhance immune-mediated wound healing, particularly to targeted macrophage through the repolarization or depletion/repopulation approaches. Generally, nanomedicine-mediated macrophage modulation is achieved by active or passive targeting. Nanoparticles can actively target macrophages by receptor-mediated endocytosis to promote the macrophage repolarization, while physicochemical properties of nanoparticles can be tuned to regulate macrophage phenotypes by passive targeting and accumulation at the infection sites as a result of phagocytosis. Small size, large surface area, and the ability to encapsulate and deliver a wide range of therapeutic cargos are the unique advantages of nanomedicine to protect the payloads in wound healing process and infiltrate quickly to wound sites for macrophage regulation in the wound treatment [45–47]. Therefore, it is interesting to investigate macrophage-targeted nanotherapeutic strategies for wound treatment.

In this review, we summarize the function of macrophages in wound immune-microenvironment and healing process. Then, we focus on strategies for engineering macrophages in details to show the advantages and disadvantages of those strategies. The cargos and physicochemical properties of the biomaterials used to modulate macrophages are discussed. We also review the role of nanomedicine in restoring the function of macrophages for wound healing. Finally, the article provides a summary of challenges and future directions of the development of macrophage-targeted nanomedicines in wound healing.

## The role of macrophages in wound immune-microenvironment and healing process

**Fig. 1:**
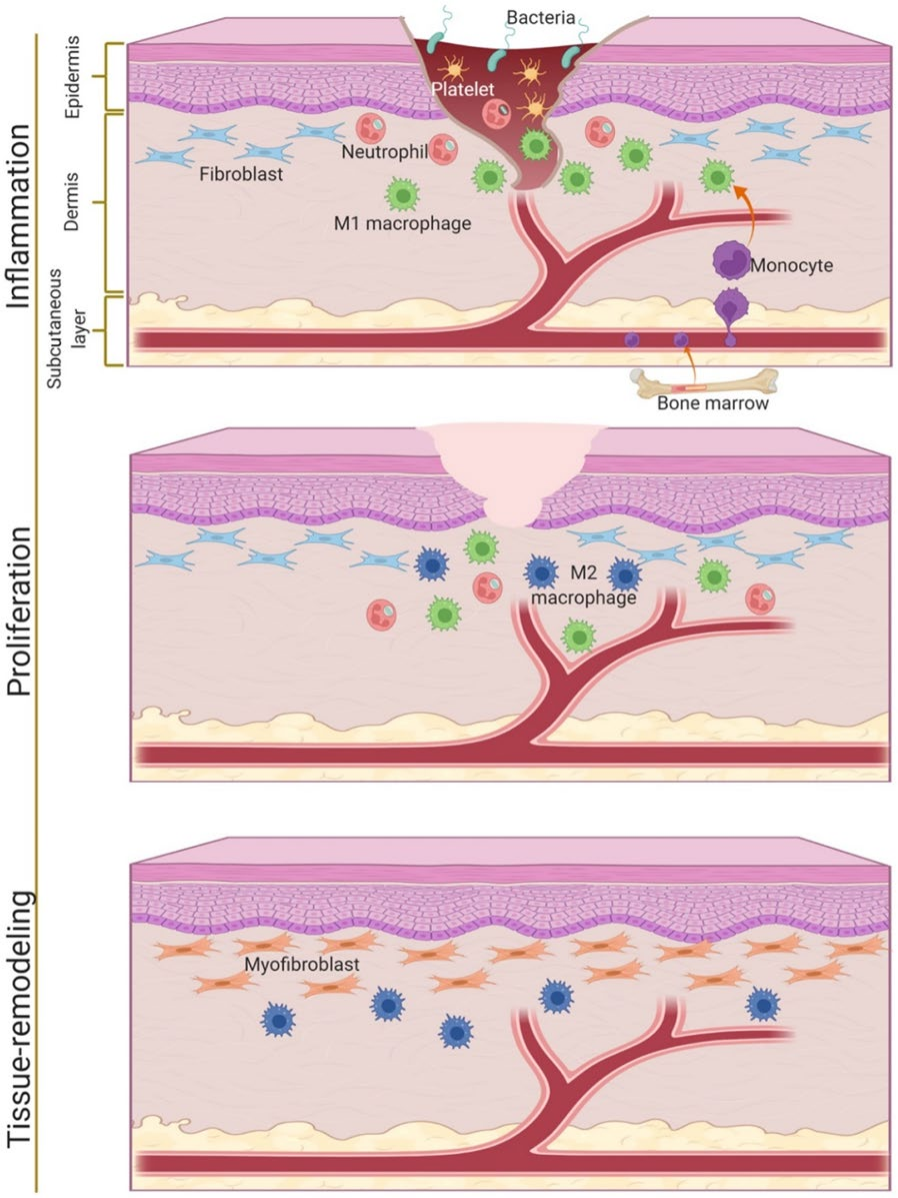
A schematic diagram of wound healing phases and mechanisms driving macrophage phenotypes in wound microenvironment

Wound immune microenvironment plays an important role in preventing pathogen invasion and tissue regeneration. Macrophages are the key immune cells for phagocytosis, regulation of inflammatory response, clearance of dead cells and pathogenic substances, and activation of tissue regeneration and remodeling. Under stimulation such as interferon gamma (IFN-γ), lipopolysaccharide (LPS), and tumor necrosis factor-α (TNF-α), M1 macrophages dominates in the early stages of wound healing. They produce pro-inflammatory mediators to foster the initial pro-inflammatory response. In addition, they involve in recruitment of other immune cells and regulate the inflammatory immune environment by interacting with T helper 1 (Th1) cells [48]. M1 macrophages are also responsible for phagocytic activity; phagocytize pathogens and foreign debris in the wound microenvironment. As wounds begin to repair, M2 macrophages are activated by interleukin-4 (IL-4) or IL-13 to generate anti-inflammatory factors, including growth factors, surface markers of scavenger receptors, IL-10, and intracellular arginase-1 (Arg-1) expression [49]. Later in the proliferation phase, M2 macrophages promote T helper 2 (Th2) responses and the adaptive immune system to provide immunomodulatory control of inflammation, which further enhance angiogenesis, fibroblast proliferation, ECM development, and re-epithelialization in the wound area [50, 51]. The M1–M2 transition prevents further damage to the wound site and guarantees the scar-free wound healing in the tissue remodeling phase. As macrophages are present throughout the entire wound recovery with dynamic changes in phenotype and function, the following sections discuss the roles of macrophages in different phases of wound healing (Fig. 1).

### Inflammation phase

The immune response is innately activated by dysfunctional cellular activities and aberrant cytokine secretion, and inflammatory cells (macrophages, neutrophils, and T-lymphacytes) are recruited from the circulation during the inflammation phase. Blood-circulating monocytes, which originate from the hematopoietic stem cells located in the bone marrow of the adult mammal, are recruited and reach the dermis layer, where they differentiate to the mature macrophages to fight pathogens simultaneously with the presence of neutrophils [17, 52]. M1 macrophages release nitric oxide (NO) to kill intracellular pathogens and stabilize the host cells [53]. Macrophages are stimulated by hypoxia to produce chemoattractants, such as chemokines, to increase the number of leukocytes and leucocytic infiltration to the wound site [24, 35, 52]. M1 macrophages are also activated to express CD86 and produce pro-inflammatory cytokines such as IL-1 and IL-6, TNF-α, and reactive oxygen species (ROS) after interaction with molecular patterns associated with pathogens, damages, and peptidoglycans released from lysed cells [48, 54, 55]. The recruitment of neutrophils (the most abundant white cells) and macrophages to the wound site lead to phagocytosis, destroying and ingestion of damaged matrix, and elimination of microorganisms and dead cells [52, 56]. M1 macrophages secreted IL-12 activate Th1 cells to initiate the adaptive immune response [48].

### Proliferation phase

To minimize further inflammation-induced tissue damage, macrophages polarize to M2 phenotype to facilitate wound healing, while inflammation subsides and the number of leukocytes reduces. IL-4 and IL-13 stimulate M2 macrophage polarization in order to secrete anti-inflammatory cytokine such as IL-10 to reduce the pro-inflammatory response, and growth factors such as vascular endothelial growth factors-α (VEGF-α), transforming growth factor-ꞵ (TGF-ꞵ), platelet derived growth factors (PDGF), and insulin like growth factor-1 (IGF-1) to boost cell proliferation and angiogenesis [24, 52]. These specific growth factors induce proliferation of fibroblasts and their differentiation into myofibroblasts to promote wound closure and collagen production, while prevent ECM degradation by up-regulation of tissue inhibitors of metalloproteinases [57–60]. Neutrophils can negatively influence tissue repair by destroying normal tissue using neutrophil proteases (e.g. elastase and cathepsin G) and free oxygen radicals (e.g. hydrogen peroxide) and delaying wound healing in the proliferation phase [52]. M2 macrophage-released IL-10 contributes to the apoptosis of neutrophils and increased collagen deposition to remove neutrophils, suppress inflammation, and enhance tissue repair [20, 52]. Subsequently, macrophages remove the apoptotic neutrophils by phagocytosis, which can prevent additional tissue damage and deposition of collagen in scar tissue [52].

### Tissue remodeling

Tissue remodeling is the final stage of wound healing. M2 macrophages generate regulatory receptors for agonist ligands of the IL-1 family and growth factors, promoting fibroblast differentiation, ECM remodeling, and angiogenesis [61]. Macrophages, along with a variety of other cell types including fibroblasts, endothelial cells, and adipocytes, produce MMP for tissue remodeling. Zinc dependent proteases MMPs (e.g. MMP-1, MMP-3, MMP-10, and MMP-12) expressed by macrophages has enzymatic activity that enhances wound healing process and ECM remodeling via restoration of the tissue morphology and the tissue function. Macrophages are responsible for breakdown of ECM fragments by secretion of MMPs, cysteine proteinases (cathepsin B and L), and serine proteases [20]. Macrophage-derived MMP-10 is critical for collagen deposition in wound healing. Type III collagen is dominant type of collagen in proliferation phase, and then is replaced by Type I collagen which is more stable in tissue remodeling phase [35]. The degradation and depositing collagen is beneficial to organize realignment of collagen networks and increase the tensile strength of tissues, which enhance regenerative capacity of the tissue [24, 35].

## Strategies for engineering macrophages

**Fig. 2:**
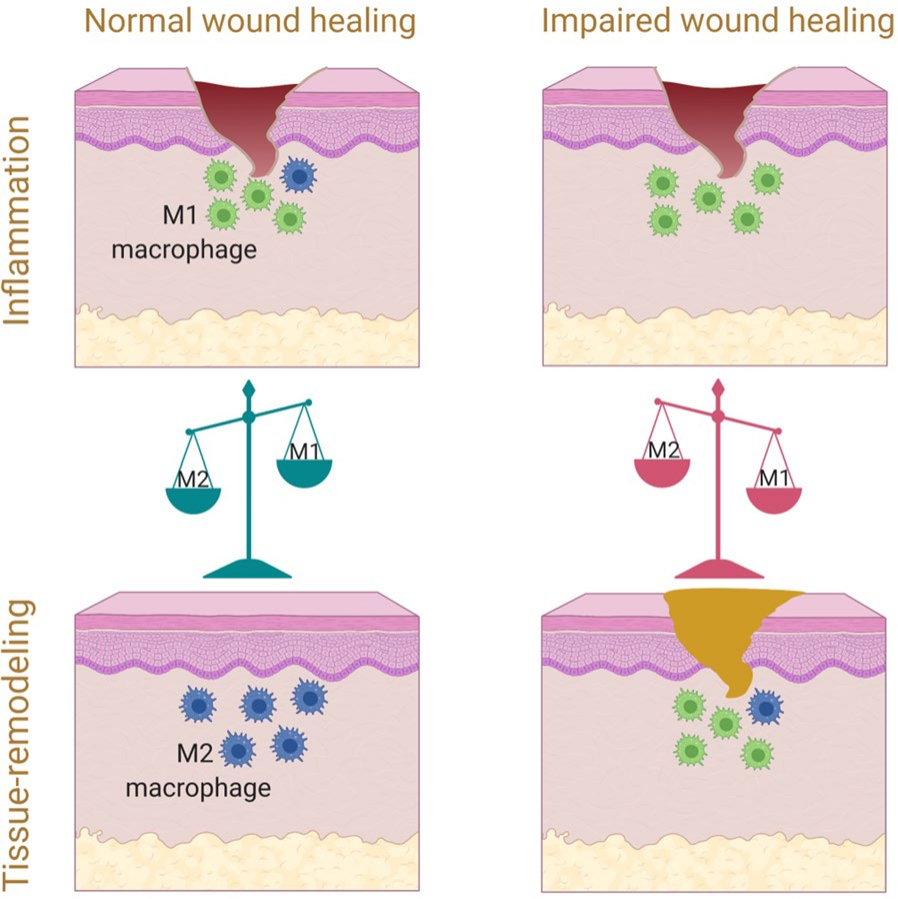
The transition of M1 to M2 phenotype in normal wound healing and chronic wounds

In chronic wound, persistent inflammation is normally observed due to reduced phagocytic capacity of macrophages and poor immunomodulation. Reduced M2 macrophage population results in a reduction of growth factor levels and changes the fine balance between pro-inflammatory and anti-inflammatory cytokines (Fig. 2). Therefore, efforts have been made to develop strategies to control inflammation and repolarize macrophages from an M1 to M2 phenotype in order to provide a suitable immune microenvironment for wound healing [62–65]. Recently, studies have reported that the depletion of M1 macrophages, increasing the amount of M2 macrophages or transition of M1 to M2 can benefit revascularization, re-epithelialization, and fibroblast regeneration especially for treatment of chronic wounds where normal shift from M1 macrophages to M2 macrophages is dysregulated [66–68].

As previously introduced, the strategies to manipulate macrophages for wound management include administration of activated macrophages, use of cytokines, dressings loaded with macrophage-modulatory therapeutic agents, and design of biomaterials or nanomedicines to manipulate macrophage infiltration or macrophage phenotypical differentiation. Although delivery of activated macrophages has been studied for decades, it is still challenging to efficiently transport activated macrophages to specific diseased sites to achieve high therapeutic benefits [69]. For example, Jetten et al. [70] reported that IL-4 or IL-10-induced M2 macrophages were found to be ineffective in the treatment of wound in wild-type mice while they delayed re-epithelialization and persistence of neutrophils in diabetic wounds. In contrast, cytokine-based strategies to balance M1–M2 macrophage phenotypes have been suggested by some studies for wound treatment [71–74]. However, disadvantages of cytokine-based therapies such as short half-life and associated systemic toxicity limited their clinical applications. Therefore, development of macrophage-modulatory biomaterials and nanomedicines has been explored as more effective strategies in wound healing [29, 69, 75]. In this review, we will mainly focus on physicochemical properties of macrophage-modulatory biomaterials and macrophage-targeted nanomedicines for wound healing.

## Physiochemical properties of immunomodulatory biomaterials

**Fig. 3:**
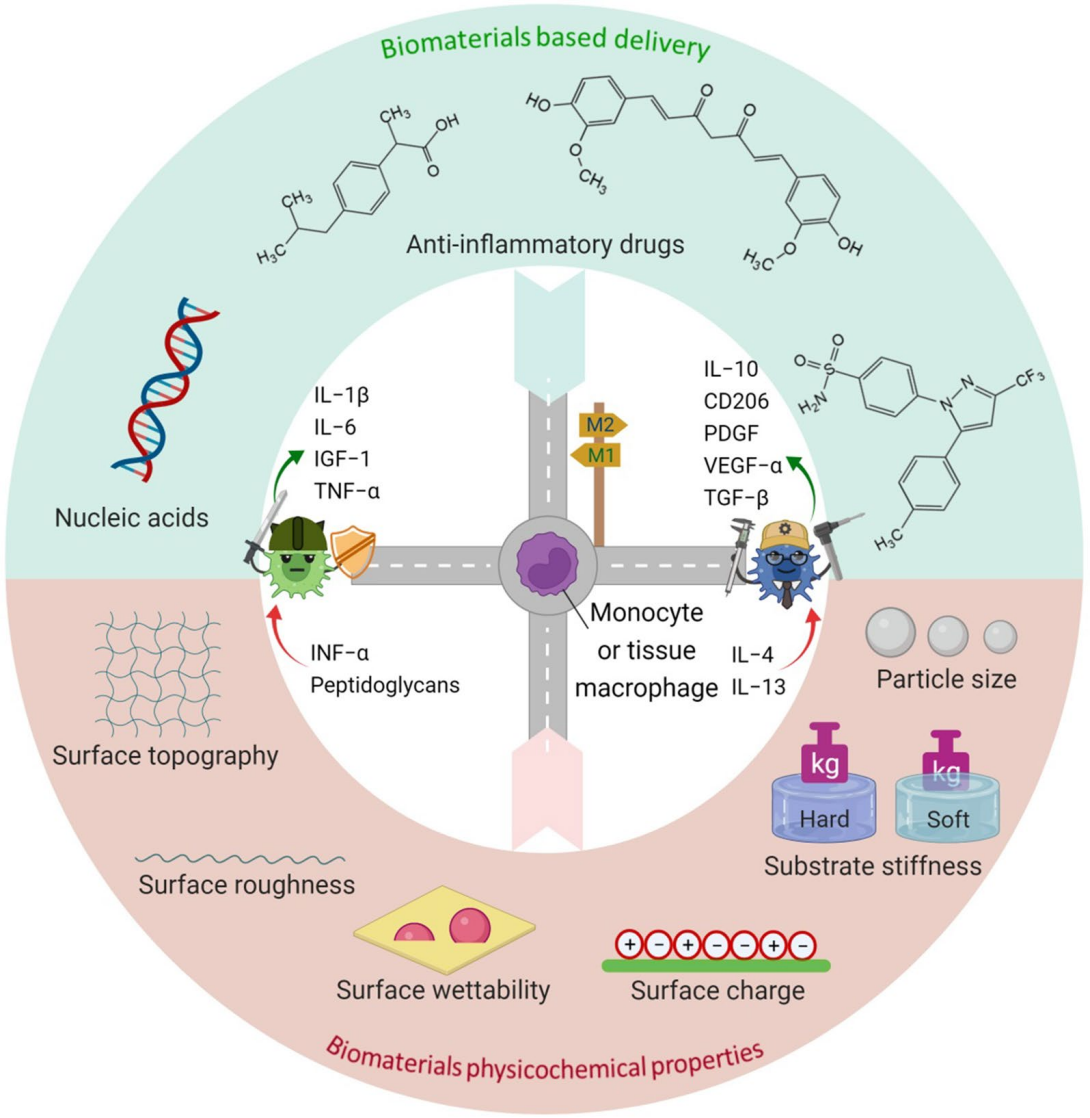
Physicochemical properties of biomaterials and biomaterials-based delivery of bioactive agents to trigger macrophage polarization

Biomaterials have attracted considerable interest as wound healing implant due to their prominent biological functions, which can provide protective environment and self-healing mechanisms after implantation of biomaterials [76–78]. Although they can initiate favorable response of the wound immune microenvironment for wound recovery, foreign body response to an implant surface may happen, dependent on interactions with the physical and chemical properties of the surface. This foreign body reaction, mainly involving macrophages as the immune cells, can lead to unwanted inflammatory response which hinders wound closure and recovery [17, 79, 80]. The impact of several physical and chemical properties such as pore size, topography, stiffness, and surface chemistry on macrophage behaviors has been investigated [19, 76, 81]. Here, the effects of the physicochemical properties on macrophage activation will be discussed in order to design better immunomodulatory biomaterials for wound regeneration. The most important physicochemical properties of biomaterials besides the biomaterials-based delivery of bioactive agents to promote macrophage polarization are illustrated in Fig. 3 and discussed in the following sections.

### Substrate stiffness

Substrate stiffness of the biomaterials has been considered as one of the most important physical features in regulating immune response. It influences macrophage functions such as motility, morphology, and polarization [82–84]. Thus, understanding molecular mechanism of substrate stiffness is beneficial for the design of immunomodulatory biomaterials in order to promote wound healing. Okamoto et al. investigated the impact of substrate stiffness on in vitro activation phenotypes of the human monocyte cell line THP-1 by comparing 1% (4 kPa), 4% (15 kPa), 10% (100 kPa) agarose gel with a plastic substrate (> 10 MPa) [82]. The results showed that 1% agarose gel substrate attenuated M1-like macrophage polarization while promoted the anti-inflammatory M2-like activation phenotype of macrophages via induced peroxisome proliferator-activated receptor γ expression. Based on a few reported studies, a recent review conducted by Davenport et al. concluded that stiffness > 100 kPa activates inflammatory behavior of macrophages and their functions during wound regeneration [85]. Li and Bratlie [86] used photopolymerization to prepare methacrylated gellan gum hydrogels with compressive moduli in the range of 5 to 30 kPa. Hydrogel with the lowest crosslinking density are expected to have the least compressive modulus. The surface of the hydrogels was coated with fibronectin to modulate macrophage proliferation, adhesion, and polarization. Phenotypic shift from pro- to anti-inflammatory was investigated and their result showed that fibronectin coated gels has no impact on M1 macrophages, while fibronectin-coated gels with the highest compressive modulus (30 kPa) promote M2 macrophages polarization, in contrast to the untreated gel. Chen et al. [87] studied the effect of the substrate stiffness of polyacrylamide hydrogels on macrophage polarization. According to this study, substrates that are similar to osteoid stiffness (60 kPa) are preferable for promoting tissue repair. The ability to detect the stiffness of substrates has an impact on macrophage polarization. Collagen fiber stiffness-like substrate (5 kPa) promoted the M1 phenotype polarization, while an osteoid stiffness-like substrate resulted in M2 phenotype polarization. In a study by Sridharan et al. [88], migration mode of macrophages was affected by changing substrate stiffness. Polyacrylamide gels with stiffness of 323 kPa impact macrophages towards a M1 phenotype by decreasing production of cytokine IL-10 and IL-6. In contrast, gels with stiffness of 11 kPa and 88 kPa prime macrophages towards M2 phenotype with an increased production of IL-10. Camarero-Espinosa et al. [89] fabricated scaffolds with various mechanical properties and stiffness using copolymer of poly(lactide-co-caprolactone) with different ratio of lactide:caprolactone monomers. It was found that scaffolds with Young’s modulus more than 40 kPa led to M2 phenotype polarization and increased secretion of IL-10 and TGF-ꞵ in vitro. Their anti-inflammatory activity also resulted in remodeling and tissue regeneration in vivo*.* On the contrary, scaffolds with Young’s modulus less than 5 kPa resulted in M1 phenotype polarization determined by production of TNF-α. Scott et al. [90] reported that macrophages derived from cord blood CD14^+^ monocytes (CB CD14^+^) were affected by substrate modulus using poly(ethylene glycol) (PEG)-based hydrogels with shear moduli of 0.1, 3.4, and 10.3 kPa. They found that substrates with shear storage modulus of 10.3 kPa activated CB CD14^+^ to anti-inflammatory phenotype macrophages. Increasing the expression of VEFG, TGF-ꞵ and CD206 showed the influence of substrate with the stiffness of 10.3 kPa on M2 macrophage polarization compared to substrates with lower stiffness. Demineralized bone matrix scaffolds with varying matrix stiffness were prepared by controlling the time of decalcification by Yao et al. [91]. The high, medium, and low stiffness scaffolds were obtained by decalcification for 1 h, 12 h, and 5 days for repairing the defected rat skull. Low matrix stiffness (0.67 MPa) induced the expression of anti-inflammatory cytokines, such as Arg-1 and IL-10, as well as polarized macrophages into M2 phenotype. These findings highlighted the importance of optimizing the mechanical stiffness to modulate macrophage response during tissue regeneration.

### Microstructure and geometry of biomaterials

Surface topography is a key physical parameter of biomaterials that may be used to modulate cellular response including migration, attachment, proliferation, and differentiation using macro-, micro-, and nano-patterning techniques [76, 83, 85, 92, 93]. Macrophages also respond to variations in surface roughness and are activated by topographic features of biomaterials, which can cause integration or failure of the implants [94]. Researchers have observed more macrophage accumulation and faster healing in vivo on implanted rough surface compared with smooth surface [95–97]. At cellular level, Barth et al. reported that RAW264.7 macrophages cultured on rough sandblasted and acid etched titanium surfaces were more likely to be activated toward anti-inflammatory M2 phenotype in healing process rather than smooth polished surface [98]. The ability of micro/nano patterned surfaces to improve macrophage adherence and polarization towards M2-like cells has been demonstrated. The effect of titanium surface roughness on human THP-1 derived macrophages was investigated by Zhang et al. [99]. The results demonstrated that low and medium rough titanium surfaces (Ra = 0.51–1.36 μm; Sa = 0.66–2.91 μm) induced the polarization of macrophages toward M2 phenotype by down-regulating the secretion and gene expression of pro-inflammatory cytokines while up-regulating the secretion and gene expression of anti-inflammatory markers compared to as-received smooth surfaces (Ra = 0.20 μm; Sa = 0.33 μm). The effect of polystyrene surface roughness on macrophage polarization was evaluated in a study by Kosoff et al. [100]. The rough micro-milled surface promoted M1 macrophage polarization, compared with the surface smoothed by acetone polishing. Therefore, modification of the surface topography is an effective approach to modulate the macrophage immune response, increase angiogenesis, and boost tissue recovery [83, 101].

In addition, the geometry of the biomaterials such as shape, size, and pore dimensions can significantly modulate macrophage behaviors. For instance, Sussman et al. examined macrophage polarization induced by subcutaneously implanted poly(2-hydroxyethyl methacrylate) hydrogels with pore size of 34 µm and 160 μm. They demonstrated that macrophages in 34 μm porous implants displayed a shift towards M1 phenotype, while macrophages immediately outside the porous structure revealed a large increase in M2 phenotypic cells [102]. In another study, the influence of pore size-induced macrophage differentiation was investigated using genipin cross-linked collagen/chitosan scaffolds with average pore size of 160 and 360 µm. The scaffolds with larger pore size induced higher degree of M1 to M2 transition and promoted macrophage secretion of anti-inflammatory and pro-angiogenic cytokines, leading to improved angiogenesis and vascularization [103]. In addition, the combinational effect of nanoporosity and chemistry on macrophage polarization into M1 and M2 phenotypes was assessed by fabricating nanoporous surfaces of controlled pore size (30, 65, and 200 nm) and lateral spacing with modified surface chemistry [104]. It is reported that macrophages on large nanoporous surfaces with acrylic acid and methyl oxazoline coatings reduced the secretion of pro-inflammatory cytokines and increased of the secretion of anti-inflammatory cytokines compared to surfaces with smaller pore sizes and methyl group rich chemistry. Camarero-Espinosa et al. demonstrated that the porosity and mechanical properties of the poly(caprolactone-co-lactide) scaffolds can influence macrophage polarization. The macrophages with a higher surface spread area polarized towards M1 phenotype, while the cells with a reduced surface spread area polarized towards M2 phenotype [89]. Moreover, the impact of nanofiber alignment on macrophage polarization was evaluated by Jia et al. [105]. Their results suggested that aligned nanofiber scaffolds significantly induced macrophage elongation and pro-healing macrophage polarization and facilitated peripheral nerve regeneration compared with random-nanofiber ones.

### Surface chemistry

Function and phenotype of macrophages can be modulated through their interactions with the chemical groups on the surface of implanted biomaterials and changing of the surface wettability [83, 92, 106]. For example, Bygd et al. [107] prepared poly(*N*-isopropylacrylamide-co-acrylic acid) nanoparticles with the average size of 600 nm and modified with various functional groups. By measuring ex vivo TNF-α, IL-10, and Arg-1:iNOS levels, it was observed that amide, alkene, ketone, and epoxide functionalization induced more M2-like phenotypes. Amide and ketone functionalized particles also showed M2 macrophage polarization in the in vivo study. Lv et al. [108] showed that hydrophilic surfaces drove macrophages into the M2 phenotype through the existence of RGD cell-binding sites in adsorbed FN and β1 attachment most likely via PI3K/Akt signaling pathway. Macrophages on hydrophobic surfaces, on the other hand, interacted with P1/P2 segments in adsorbed FG through 2 attachments, resulting in the development of the M1 phenotype, most likely by NF-B activation. Later, Visalakshan et al. [109] studied the role of surface chemistry and wettability on macrophage response. Hydrophilic surfaces had a higher adsorption of dysopsonin albumin, leading to greater expression of anti-inflammatory cytokines by macrophages. Contrary, surface hydrophobicity caused higher opsonin IgG2 adsorption and increased production of pro-inflammatory signaling molecules. In another study, macrophage polarization on three different substrates including super-hydrophilic nanotubular Ti surfaces, air-annealed nanotubular Ti surfaces, and pure Ti substrates was evaluated. The results showed that super-hydrophilic nanotubular Ti surfaces increased expression of M2 markers (IL-10 and TGF-ꞵ) and down-regulated expression of M1 markers [110]. Kosoff et al. reported the combinational effects of surface topography and hydrophilicity on macrophage differentiation [100]. For macrophages culture on smooth surfaces, an increase in surface hydrophilicity led to increased expression of two of the three M2-associated genes evaluated, while for macrophages cultured on rough surfaces, an increase in hydrophilicity resulted in significant increases in two of the three M1-associated genes and a significant increase in only one of the M2-associated genes. Perez-Calixto et al. [111] studied how surface grafting of polypropylene and polytetrafluoroethylene with amino groups modulated macrophage polarization. They found that amino-functionalization reduces pro-inflammatory cytokine release, which could benefit wound healing. The effect of electrospun silk fibroin-silk sericin fibrous films on macrophage polarization and vascularization was assessed in a study by Wang et al. [112]. The addition of silk sericin stimulated macrophages to release more M1 and M2 cytokines. When the ratio of silk fibroin:silk sericin in electrospun films reached 7:3, the hydrophilicity enhanced, resulting in the higher ratio of M2/M1 phenotypes and the highest degree of vascularization.

In summary, the hydrophilicity or wettability of the substrate has been considered as a key feature of surface properties that influences macrophage adhesion and polarization. It has been demonstrated that increasing the hydrophilicity or wettability could up-regulate the anti-inflammatory cytokines. Design of immunomodulatory biomaterials to modulate macrophages phenotype and number are of great potential in wound tissue regeneration. To achieve successful clinical approaches, studying the behavior of macrophages in contact with biomaterials interface, optimization of physical parameters of biomaterials particularly topography, and deep understanding of the surface chemistry by employment of ions and molecules with different electrical charge density are needed in preparation of new generation of biomaterials.

## Nanomedicines targeting macrophages for wound healing

Nanomaterials have been considered as a promising approach for promoting the wound regeneration due to their superior physicochemical properties, excellent drug loading capacity, and biocompatibility [113, 114]. During the last few decades, researchers have explored various types of organic or inorganic nanomaterials to be used in the development of wound dressing materials to act as antibacterial agents and stimulate wound healing process by providing sustained drug release, mimic the properties of ECM, and regulate cell behaviors [113, 115]. Particularly, nanomaterials can modulate the macrophages in the wounds via macrophage depletion or phenotype repolarization. The nanomaterials-based strategies to modulate macrophages in the wound healing application have been explained in the following subsections and summarized in Table 1.

**Table 1:** Classification of nanoparticles-based materials for delivery of relevant payloads to modulate macrophage phenotypes in the wound healing process

Type of nanoparticles	Payload	In vitro/in vivo model	Pathways	Therapeutic strategies	References
Drug-free inorganic nanomaterials induce macrophage polarization					
BGNPs	–	In vivo	Decreasing the inflammatory cytokines and increasing the secretion of anti-inflammatory cytokines	Polarization of M1 to M2 phenotype	[116]
BGNPs	–	In vivo	Macrophage proliferation and polarization toward M2 phenotype to facilitate wound closure and re-epithelialization in diabetic wounds	Polarization of M1 to M2 phenotype	[117]
Gold-mesoporous BGNPs	–	In vivo	Decreasing the inflammatory cytokines and increasing the secretion of anti-inflammatory cytokines	Polarization of M1 to M2 phenotype	[118]
Ceria nanocrystals decorated MSNPs	–	In vivo	Reducing ROS, differentiation of monocytes to macrophages, and modulation of anti-inflammatory factors	Polarization of M1 to M2 phenotype	[119]
Drug-free organic nanomaterials induce macrophage polarization					
Nanofibrous scaffolds including copolymer of poly(lactide-co-caprolactone) and heart decellularized ECM	–	In vivo	Collagen deposition and decreasing the inflammatory cytokines and increasing the secretion of anti-inflammatory cytokines	Polarization of M1 to M2 phenotype	[122]
Thioether grafted hyaluronic acid nanofibrous	–	In vivo	Decreasing the inflammatory cytokines and increasing the secretion of anti-inflammatory cytokines	Polarization of M1 to M2 phenotype	[123]
Coaxial nanofibers of PLGA/fibrinogen and PLGA/collagen	–	In vivo	Promotion of the secretion of immunosuppressive factors as well as wound healing growth factors	Polarization of M1 to M2 phenotype	[124]
α-Gal epitope nanoparticles	–	In vivo	Decreasing the inflammatory cytokines and increasing the secretion of anti-inflammatory cytokines	Polarization of M1 to M2 phenotype	[125]
Supramolecular peptide hydrogel nanoparticles	–	In vitro	Reduction of NO and decreasing the inflammatory cytokines and increasing the secretion of anti-inflammatory cytokines	Polarization of M1 to M2 phenotype	[126]
Amphiphilic galactomannan nanoparticles	–	In vivo	Decreasing the inflammatory cytokines and increasing the secretion of anti-inflammatory cytokines	Polarization of M1 to M2 phenotype	[127]
Drug-free organic–inorganic hybrid nanomaterials induce macrophage polarization					
Metallic AgNPs–collagen/chitosan scaffold	–	In vivo	Promotion of the inflammatory cytokines and increasing the secretion of anti-inflammatory cytokines	Polarization of M1 to M2 phenotype	[130]
Magnesium particles embedded in electrospun PCL nanofibers	–	In vivo	Collagen deposition and decreasing the inflammatory cytokines and increasing the secretion of anti-inflammatory cytokines	Polarization of M1 to M2 phenotype	[131]
SiO_2_ nanoparticles were modified by konjac glucomannan	–	In vivo	Collagen deposition and decreasing the inflammatory cytokines and increasing the secretion of anti-inflammatory cytokines	Polarization of M1 to M2 phenotype	[132]
AgNPs was decorated with zwitterionic poly(carboxybetaine-co-dopamine methacrylamide) copolymer	–	In vivo	Acceleration of the migration of fibroblast cells, factors for pro-inflammatory level increased as well as markers for macrophage activity	Polarization of M1 to M2 phenotype	[133]
Inorganic/organic hybrid nanocomposites of silver/talc nanoparticles coated with chitosan	–	In vivo	Increasing vascularization and angiogenesis, collagen deposition and decreasing the inflammatory cytokines and increasing the secretion of anti-inflammatory cytokines	Polarization of M1 to M2 phenotype	[134]
Gold/perlite mesoporous nanocomposites coated with chitosan	–	In vivo	Decreasing the inflammatory cytokines and increasing the secretion of anti-inflammatory cytokines	Polarization of M1 to M2 phenotype	[135]
Magnesium-containing BGNPs incorporated with hyaluronic acid and quaternized chitosan hydrogels	–	In vivo	Increasing vascularization and angiogenesis, collagen deposition and decreasing the inflammatory cytokines and increasing the secretion of anti-inflammatory cytokines	Polarization of M1 to M2 phenotype	[136]
Drug-loaded inorganic nanomaterials induce macrophage polarization					
AgNPs	Tannic acid	In vivo	Promotion of epithelialization, angiogenesis, and granulation tissue by increasing the expression of anti-inflammatory cytokines	Polarization of M1 to M2 phenotype	[137]
Super paramagnetic iron oxide nanoparticles	Heparin bonded fibroblast growth factor	In vivo	Controlled release of fibroblast growth factor. Granulation formation and collagen deposition due to the promotion of cell proliferation and M2 phenotype polarization	Polarization of M1 to M2 phenotype	[138]
AuNPs	Snail mucus (Helix Aspersa)	In vitro	Reduction of LPS induced IL-6 and IL-1ꞵ cytokine levels and elimination of iNOS synthesis	Depletion of M1 phenotype	[139]
Drug-loaded organic nanomaterials induce macrophage polarization					
Silk nanofiber	Asiaticoside	In vivo	Regulation of inflammatory reaction and vascularization	Polarization of M1 to M2 phenotype	[140]
Hyaluronic acid nanoparticles	miR-223	In vivo	Increasing anti-inflammatory gene and decreasing pro-inflammatory markers	Polarization of M1 to M2 phenotype	[141]
Phenyl boronic acid-modified alginate nanocapsules	Amikacin and naproxen	In vivo	Decreasing the inflammatory cytokines and increasing the secretion of anti-inflammatory cytokines	Polarization of M1 to M2 phenotype	[142]
Membrane with nanotopography of dihydroxyterephthaldehyde and 5,10,15,20-(tetra-4-aminophenyl)porphyrin	Ibuprofen	In vivo	Reducing the inflammatory reaction of macrophages and increasing the proportion of M2 macrophages at the injury site	Polarization of M1 to M2 phenotype	[143]
Phosphatidylserine-nanoliposomes	Apoptotic cell	In vivo	Increasing the related cytokines to M2 macrophages, expression of the vascular endothelial marker CD31and accelerate wound closure	Polarization of M1 to M2 phenotype	[144]
Drug-loaded organic–inorganic hybrid nanomaterials induce macrophage polarization					
MSNPs coated with collagen	Gentamicin and rifamycin	In vivo	Decreasing the inflammatory cytokines and increasing the secretion of anti-inflammatory cytokines	Polarization of M1 to M2 phenotype	[147]
Mesoporous silica coated AgNPs in poloxamer hydrogel	Gentamicin	In vivo	decreasing the related cytokines to M1 macrophages, expression of the marker CD86 and accelerate diabetic wound healing	Depletion of M1 phenotype	[148]
MSNPs coated with cellulose acetate	Econazole nitrate and triamcinolone acetonide	In vivo	Decreasing the inflammatory cytokines and increasing the secretion of anti-inflammatory cytokines	Polarization of M1 to M2 phenotype	[149]

### Drug-free inorganic nanomaterials induce macrophage polarization

Inorganic nanomaterials have been used to regulate macrophage polarization because inorganic materials (e.g. metal ions) are able to improve inflammatory microenvironment and wound recovery [106]. Dong et al. studied the mechanism of bioactive glass (BG) enhancing wound healing via macrophage regulation [116]. They found that BG ionic products activated macrophages towards the M2 phenotype and stimulated macrophages to reduce inflammation and wound closure compared to control. Later, Xie et al. [117] investigated the effects of bioactive glass nanoparticles (BGNPs) on macrophage proliferation, migration, and polarization for diabetic wound healing. The M1-to-M2 macrophage phenotype switch was observed after treatment of BGNPs with the concentration 20 µg/ml, whereas higher concentration of the particles (100 µg/ml) caused prolonged M1 macrophage polarization and decelerated wound healing. The BGNPs regulated macrophage polarization, wound closure, and re-epithelialization in diabetic wounds in a dose-dependent manner. In addition, the influences of metal conjugated BG materials on wound healing were also investigated experimentally in a range of studies. Marza et al. [118] prepared ointment formulations with BG conjugated gold nanoparticles (AuNPs) mixed with Vaseline to repair the skin wound. Furthermore, Wu et al. [119] has developed a highly versatile ROS-scavenging tissue adhesive nanocomposite using ceria nanocrystals-decorated mesoporous silica nanoparticles (MSNPs). A significant decrease in the local inflammatory response was observed in ceria nanocrystals-decorated MSNPs treated rats by staining the infiltration of CD68-positive macrophages at day 5 post-wounding; suggesting this formulation efficiently accelerated the wound healing and limited scar formation.

### Drug-free organic nanomaterials induce macrophage polarization

Organic nanomaterials, particularly the polymeric nanostructures, have been widely used for wound healing because of relatively simple fabrication methods, versatile surface functionalization process, biodegradability, and biocompatibility. The researchers discovered that some polymeric nanostructures can improve macrophage activation while also effectively boosting angiogenesis and re-epithelialization in the wound healing process [120, 121]. Kim et al. [122] fabricated nanofibrous electrospun hybrid scaffold using copolymer of poly(lactide-co-caprolactone) and decellularized heart tissue. By analyzing macrophage population and their immune response, the results showed that the scaffolds had anti-inflammatory effects by increasing initial M2 macrophages. In addition, the scaffolds reduced scarring by rapid replacement of collagen type III to collagen type I after 21 days post-surgery, evaluated by the immunohistochemistry studies. Liu et al. [123] synthesized electrospun thioether grafted hyaluronic acid nanofibrous hydrogels to promote macrophage modulation for diabetic wound healing. It was observed that the thioether grafted hyaluronic acid nanofibers scavenged the ROS, reduced the inflammatory response, promoted the macrophage polarization from M1 to M2 phenotype, leading to improved wound healing phase transition, compared with the control group. ECM-biomimetic coaxial nanofibrous scaffolds made of poly(lactic-co-glycolic acid) PLGA/fibrinogen as the shell and PLGA/collagen as the core were prepared for the repair of chronic wounds by Sun et al. [124]. The biomimetic coaxial scaffolds promoted adipose-derived mesenchymal stromal cells to secrete immunosuppressive factors (COX-2 and TSG-6) as well as wound healing growth factors (TGF-ꞵ and VEGF-α). The secretion of immunosuppressive factor modulated the macrophage phenotypic switch from M1 to M2. In vivo study in rat models exhibited the biomimetic coaxial scaffolds reduced inflammation and consequently accelerated diabetic wound healing. The α-gal nanoparticles with multiple α-gal epitopes as carbohydrate antigen were prepared and their effects on diabetic wound treatment were assessed. These nanoparticles were able to recruit M2 macrophages, which release pro-healing cytokines/growth factors to accelerate wound healing [125]. More recently, injectable hybrid supramolecular hydrogels were synthesized by electrostatic interactions between phosphorylated tripeptide and vinylimidazole and ketoprofen polymeric nanoparticles [126]. It was demonstrated that the hybrid hydrogels activated anti-inflammatory macrophages by causing significant reduction of NO. In a study by Peled et al. [127], amphiphilic nanoparticles were produced by the self-assembly of a copolymer of hydrolyzed galactomannan, a natural polysaccharide of galactose and mannose, grafted with poly(methyl metacrylate). By measuring M1 and M2 markers, the results suggested that these nanoparticles can polarize towards the M2-like phenotype. They also found that synthesized grafted amphiphilic nanoparticles were able to accelerate wound healing process.

### Drug-free organic–inorganic hybrid nanomaterials induce macrophage polarization

As discussed, some inorganic nanoparticles possess antimicrobial, immunomodulatory, and wound healing properties by regulating cells, cytokines, and growth factors, while certain organic nanomaterials can benefit tissue regeneration by activating healing-related immune response and facilitating ECM remodeling. Therefore, researchers have incorporated inorganic nanoparticles with organic coating or matrix to form organic–inorganic hybrid nanomaterials. Recently, they have been attracted increasing attentions to be used in macrophage-modulatory wound tissue engineering [128, 129]. For instance, metallic silver nanoparticles (AgNPs) conjugated collagen/chitosan hybrid scaffold was fabricated and its therapeutic potential to improve wound healing was investigated [130]. They demonstrated that hybrid scaffold attenuated inflammatory response by regulating macrophage activation and normalized the wound healing process in rat models. The results suggested that hybrid scaffold was antibacterial, anti-inflammatory, and enhanced wound healing by modulation of macrophage activation and fibroblast migration. Adhikari et al. [131] incorporated magnesium particles in electrospun polycaprolactone nanofibers and then assessed their influence on macrophage infiltration and polarization, cytotoxicity, and collagen deposition. The animal study showed that the magnesium-loaded nanofiber mesh resulted in the presence of M2-like, reparative macrophages, well vascularization, and improved healing within 28 days compared with the mesh alone sample. Gan et al. [132], modified SiO_2_ nanoparticles with konjac glucomannan to enhance diabetic wound healing via reprogramming the murine bone marrow-derived macrophages. The modified SiO_2_ induced the formation of M2-like macrophages by clustering mannose receptor on the cells. Subsequently, the activated macrophages secreted the cytokines, leading to fibroblast proliferation and ECM secretion. These results suggested that modified SiO_2_ display great therapeutic potential for cutaneous wounds by effectively suppressing excessive or persistent inflammation and fibrosis. More recently, Xiang et al. [133] demonstrated that mussel-inspired zwitterionic AgNPs decorated with poly(carboxybetaine-co-dopamine methacrylamide) copolymer reduced macrophage-mediated inflammatory response during wound management. The AgNPs decorated copolymer suppressed the expression of pro-inflammatory cytokines and inhibited CD68 macrophage activation, leading to improved wound healing. In addition, Daghian et al. [134] fabricated inorganic/organic hybrid nanocomposites using silver/talc nanoparticles and their chitosan-capped derivatives. The influence of these nanocomposites on biocompatibility, anti-oxidant, antibacterial functions, and macrophage polarization in wound healing process was evaluated. In addition to controlling of bacterial infection, the hybrid nanocomposites activated M2 macrophages and increased the secretion of IL-10, CD206, Arg-1, fibroblast growth factor (bFGF), and collagen type I, which further resulted in fibroblast migration and wound closure. They demonstrated that these hybrid nanocomposites can provide a safe and effective strategy for infected wound healing. Gharehpapagh et al. [135] also reached a similar conclusion by developing AuNPs/perlite mesoporous nanocomposites with *Urtica dioica* extract and its chitosan-capped derivatives. It was found that the hybrid nanocomposites induced the M2 macrophages polarization, evidenced by the high expression of IL-10, bFGF, and Arg-1. They showed that the hybrid nanocomposites accelerated treatment of infected wounds through inhibiting bacterial infection and macrophage-based healing. Zhu et al. [136] prepared nanocomposite hydrogel dressings by incorporating magnesium-containing BGs into hydrogel formed by hyaluronic acid-modified phenyl boronate acid and quaternized chitosan. By analyzing the expression of pro-inflammation and anti-inflammation genes, it was reported that magnesium in the nanocomposite hydrogels suppressed inflammation. This modified hydrogel accelerated wound healing in diabetic rat models by improving granulation tissues formation, vascularization, collagen deposition, the formation of blood vessels, and decreasing inflammation in the wound sites.

### Drug-loaded inorganic nanomaterials induce macrophage polarization

Inorganic nanomaterials can also be functionalized and conjugated with anti-inflammatory molecules as drug carriers for macrophage regulation in wound regeneration. Taking the advantage of antioxidant, antimicrobial, and anti-inflammatory properties of tannic acid, Orlowski et al. [137], synthesized tannic acid-modified AgNPs and evaluated their wound healing properties. Tannic acid-modified AgNPs showed an advantageous profile of pro-inflammatory cytokines produced by macrophages compared with control, which improved immune microenvironment to promote keratinocyte and fibroblast migration, proliferation, and angiogenesis. Wu et al. [138] developed bFGF-loaded dopamine-heparin-conjugated Fe_3_O_4_ magnetic nanoparticles (bFGF-HDC@Fe_3_O_4_) for enhanced wound repair. The expression of anti-inflammatory cytokines (Arg-1, IL-10, and CD206) increased, while the expression of pro-inflammatory cytokine (iNOS and TNF-α) decreased in the presence of heparin [138]. bFGF-HDC@Fe_3_O_4_ stabilized and released bFGF in a sustained manner. Both in vitro and in vivo evaluation showed bFGF-HDC@Fe_3_O_4_ induced macrophage polarization toward anti-inflammatory M2 phenotypes. The promoted cell proliferation and macrophage polarization further benefited the wound regeneration including granulation formation and collagen deposition. Snail slime from *Helix aspersa* can promote cell regeneration and growth, while inhibit inflammation. Thus, Gubitosa et al. [139] synthesized AuNPs with snail slime for wound healing application. Polarization of RAW264.7 macrophages into anti-inflammatory phenotypes was evaluated. They found that AuNPs conjugated with snail slime can modulate the inflammatory response induced by LPS in murine macrophages by significantly reducing the levels of IL-1β, IL-6, and iNOS.

### Drug-loaded organic nanomaterials induce macrophage polarization

Organic nanomaterials can protect the therapeutic agents from degradation and exhibit sustained release of incorporated drugs, as well as overcome the limitations of conventional dressings such as bleeding, tissue damage, and preparation issues. Therefore, researchers have applied organic nanomaterials as delivery vehicles for controlled release of bioactive molecules which can regulate macrophage phenotypic switch for wound recovery. Liu et al. [140] loaded asiaticoside as an anti-inflammation and antioxidant drug in silk nanofiber hydrogels to stimulate collagen synthesis and promote angiogenesis for skin regeneration. Both the in vitro and in vivo studies demonstrated that the asiaticoside-laden hydrogel matrices regulated inflammatory reaction and vascularization towards scarless tissue regeneration. After treatment with the asiaticoside-laden hydrogel matrices, highest ratio of M2/M1 macrophages was reported during remodeling compared with other treatment groups. It was also observed that the asiaticoside-laden hydrogel caused a lower ratio of collagen type I to collagen type III and the deposited collagen microstructure, confirming scarless wound recovery. Saleh et al. [141], proposed an adhesive hydrogels containing miR-233 microRNA-loaded hyaluronic acid nanoparticles to polarize macrophages into anti-inflammatory phenotypes for wound healing. The polarization of macrophages to the M2 phenotype resulted from miR-233-loaded hydrogel treatment was observed using cellular and animal models. This immune response also induced the formation of uniform vascularized skin at the wound site and accelerated wound healing. Hu et al. [142] developed a smart pH- and ROS-responsive injectable hydrogel with self-healing and remodeling capability using phenyl boronic acid-grafted alginate to control the delivery of antibiotic amikacin and anti-inflammatory drug naproxen for wound healing. These drug-loaded micelle hydrogels inhibited the inflammatory response of macrophages by decreasing the expression of TNF-α while increased the expression of IL-10 after tropical administration, leading to promising healing outcome at the infected wound area. Ding et al. [143] reported an ibuprofen-encapsulated porphyrin-covalent organic framework-based membrane (IBU@DhaTph-membrane). They demonstrated that IBU@DhaTph-membrane dressing had excellent anti-infection and tissue remodeling activities by increasing local M2 macrophages and M2/M1 ratio, neovascularization and granulation tissue area, and collagen deposition. A study by Zhang et al. [144] explored the anti-inflammatory and pro-healing effects of apoptotic-cell-inspired deformable phosphatidylserine-containing nanoliposomes on diabetic chronic wounds. The prepared system persistently bound to macrophage membranes and efficiently induced M2-like macrophage polarization, resulting in improved anti-inflammatory and pro-healing responses of macrophages, increased vascularization, and accelerated wound closure.

### Drug-loaded organic–inorganic hybrid nanomaterials induce macrophage polarization

Organic–inorganic hybrid nanocomposites are a favorable group of nanomaterials with great potentials in macrophage regulation for wound management due to their specific characteristics, including drug-loading, anti-inflammatory, antimicrobial, proangiogenic, proliferative, and remodeling properties [145, 146]. For intense, Mebert et al. [147] combined core–shell silica particles loaded with gentamicin sulfate and sodium rifamycin with concentrated collagen type I hydrogels to form a drug-loaded collagen-silica nanocomposites. These nanocomposites were able to provide prolonged release of two topical antibiotics, while caused the absence of M1 inflammatory macrophages in the wound bed and solved infection-triggered inflammation. This study illustrated the therapeutic potential of the collagen-silica nanocomposites to prevent infection and promote cutaneous wound repair. Wang et al. [148] fabricated biomimic virus-like mesoporous silica coated Ag nanocubes loaded with gentamicin in a hydrogel dressing for bacteria infected diabetic wound healing. The prepared coating allowed successful delivery of gentamicin and effective bacteria adhesion, leading to improved antibacterial activity. Wound immunohistochemical staining of CD86 was used to detect the pro-inflammatory M1 macrophage marker. The CD86 positive cells in gentamicin hydrogel treated group was higher than those of mesoporous silica coated AgNPs loaded with gentamicin hydrogel treated group. Depletion of M1 macrophages led to improve diabetic wound healing. In a similar study by Maheen et al. [149], econazole nitrate and triamcinolone acetonide was loaded in MSNPs and coated with cellulose acetate. The improved efficiency of econazole nitrate and triamcinolone acetonide loaded MSNPs was associated with controlled drug delivery at the wound site, reduced toxicity of the therapeutic agents by their encapsulation, and macrophage infiltration and polarization during 2 weeks wound healing process. Therefore, the skin tissue of these nanoparticles treated group developed faster than the free drug control groups.

## Conclusion and future perspective

Regulation of macrophages is an important process during the wound development and healing. Macrophages differentiate into M1 phenotype in the early phase of wound healing with production of inflammatory factors and cytokines to phagocytize and destroy foreign bodies and necrotic tissues. Macrophages in normal wound can be effectively transitioned from the M1 phenotype in the inflammatory phase to the M2 phenotype in the proliferation and tissue remodeling phase, allowing them to dominate healing process and complete the wound repair. However, in chronic or infected wounds, it is challenging to induce the polarization of M1 macrophages to M2 phenotype in the proliferation and tissue remodeling phase, leading to impaired tissue repair.

Nanomaterials have been developed to achieve macrophage polarization as well as control of infections via controlled delivery of molecules to alter macrophage number or promote the transition of macrophage phenotypes, direct delivery of activated macrophages, delivery of cytokines, and design of biomaterials to manipulate the macrophage phenotype differentiation. Large specific area, controllable size and porosity, and loading capability are the main advantages of nanomaterials in biomedical applications, particularly wound treatment. Generally, physicochemical properties of nanomaterials, including hydrophilicity of biomaterials surfaces, surface topography, and substrate stiffness play a critical role in modulating macrophage phenotypes. Evidences suggest that smooth and hydrophobic surface, and soft substrate are associated with M1 phenotype polarization, while hydrophilic and rough surface and harder substrate can regulate macrophages toward the M2 phenotype polarization.

The interaction of biomaterials and macrophages have been investigated and showed their potentials in regulation of macrophage polarization. However, more research is needed in order to provide better understanding for preparation of engineered biomaterials as well as to overcome the remaining challenges related to immune regulation during different phases of wound healing. In addition, it is difficult to maintain the biological function of the biomaterials as degradation of biomaterials is one of the most important issue during wound treatment. There is limited information related to the degradation profile of the implant, the biocompatibility of both initial materials and degraded products, and dynamic function of materials during degradation in the complex biological environment. Therefore, comprehensive investigation of the wound dressing materials and degraded products to induce macrophage polarization is crucial for clinical success and patient safety.
